# 580. Hesitancy in Uptake and Recommendation of COVID-19 Vaccines by US Healthcare Workers

**DOI:** 10.1093/ofid/ofab466.778

**Published:** 2021-12-04

**Authors:** Steven S Spires, Rebecca Rayburn-Reeves, Elizabeth Dodds Ashley, Jenna Clark, Avani P Desai, Jan Lindemans

**Affiliations:** 1 Duke University School of Medicine, Durham, North Carolina; 2 Duke University, Durham, North Carolina; 3 Duke Center for Antimicrobial Stewardship and Infection Prevention, Durham, NC

## Abstract

**Background:**

The COVID-19 pandemic has brought vaccination to the forefront of discourse on public health. The rapid speed of COVID-19 vaccine development, utilization of novel technology, and an atmosphere of politicized misinformation have created a perfect storm for vaccine hesitancy. As early adopters of vaccination, HCWs set an example for the general population; as trusted sources of medical information, they educate and inform. However, comparatively little work has investigated HCWs' attitudes toward vaccination and how those attitudes drive their recommendation behavior.

**Methods:**

We surveyed hospital employees about their personal reasons for hesitancy and beliefs about patient hesitancies and randomly assigned them to see one of three messages aimed at increasing vaccine confidence. Message themes included an appeal to return to normal life (Normalcy), a risk comparison between vaccinating or not (SDT), and an explanation of the speed of safe and effective vaccine development (Process).

**Results:**

Of the 674 NC hospital employees who completed our survey in February 2021, 98% had been offered the COVID-19 vaccine, and 80% had already accepted. For the 20% who had not received the vaccine, the top reasons for hesitancy involved the speed of development and testing, and concerns of vaccine safety and effectiveness. We also found differences in susceptibility to misinformation and vaccine hesitancy across political affiliation, which was higher in Republicans compared to Democrats. HCWs were generally very comfortable recommending the COVID-19 vaccine to patients and supported the idea of sharing the message they read. Although the risk comparison message was most trusted personally, the process message was rated as both the most helpful to patients and the most likely to be shared with them (see Figure 1). This suggests that what is most appealing on a personal level is not necessarily what a HCW would recommend to their patients.

Rating of personal opinions of the passages.

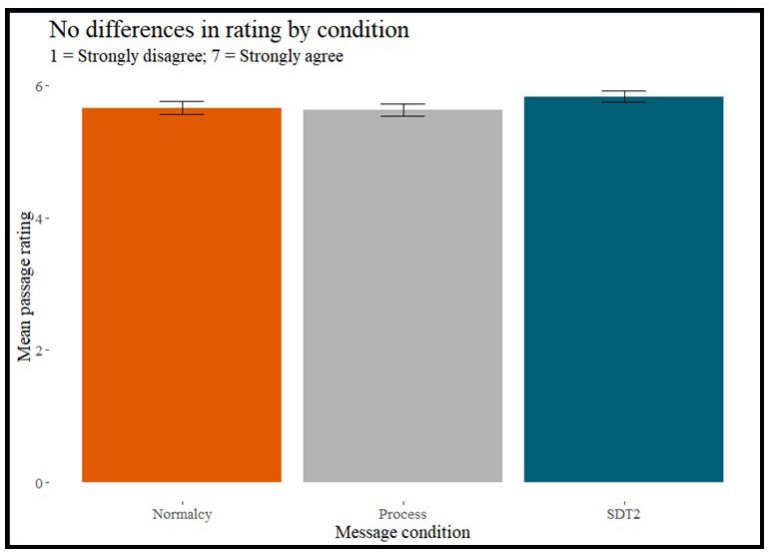

On a scale from 1 to 7 with 1 = Strongly Disagree and 7 = Strongly Agree. This chart shows the average message ratings across the board when answering whether they thought the passages were understandable, helpful, correct, believable, and trustworthy. (Error bars are 95% CI) There was no significant difference across the messages. The Process message is seen as most helpful and is most likely to be shared with patient than the other messages

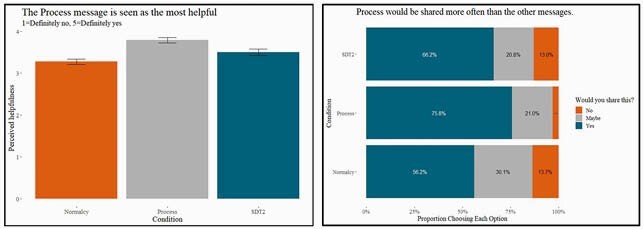

On left, the average answer on a scale from 1 to 5 for “Do you think the passage you just read would help your patients feel more comfortable about getting the vaccine?” and on right, the average answer for “Would you share this passage with your patients?”

**Conclusion:**

HCWs' high uptake and minimal hesitancy in recommending the COVID-19 vaccine is encouraging and merits further exploration for how to increase confidence in HCW who are hesitant to discuss and recommend vaccines to patients, as several highlighted the importance of respecting patient autonomy.

**Disclosures:**

**Rebecca Rayburn-Reeves, PhD**, **Centene Corporation** (Grant/Research Support, Research Grant or Support) **Jenna Clark, PhD**, **Centene Corporation** (Grant/Research Support, Research Grant or Support) **Jan Lindemans, PhD**, **Centene Corportation** (Grant/Research Support, Scientific Research Study Investigator)

